# Effect of Soybean Protein Concentrate Preparation on Copy Numbers and Structural Characteristics of DNA from Genetically Modified Soybean

**DOI:** 10.3390/foods12102031

**Published:** 2023-05-17

**Authors:** Yan Du, Fusheng Chen, Kunlun Liu, Chen Chen

**Affiliations:** 1 College of Food Science and Engineering, Henan University of Technology, Zhengzhou 450001, China; yandu@haut.edu.cn (Y.D.); knlnliu@126.com (K.L.); 15515658204@163.com (C.C.); 2 National Engineering Research Center of Wheat and Corn Further Processing, Henan University of Technology, Zhengzhou 450001, China; 3 College of Food Science and Nutritional Engineering, China Agricultural University, Beijing 100083, China

**Keywords:** genetically modified soybean, soybean protein concentrate, DNA degradation, copy numbers, structural characteristics

## Abstract

To regulate the degradation of transgenic DNA and lay theoretical foundations for the rational utilization of genetically modified (GM) products, variations in copy numbers and structural characteristics of DNA from GM soybean event GTS 40-3-2 during soybean protein concentrate (SPC) preparation were evaluated. Results showed that defatting and the first ethanol extraction were key procedures inducing DNA degradation. After these two procedures, copy numbers of the *lectin* and *cp*4 *epsps* targets decreased by more than 4 × 10^8^, occupying 36.88–49.30% of the total copy numbers from raw soybean. Atomic force microscopy images visually revealed the degradation of DNA that thinned and shortened during SPC preparation. Circular dichroism spectra suggested a lower helicity of DNA from defatted soybean kernel flour and a conformation transition of DNA from B-type to A-type after ethanol extraction. The fluorescence intensity of DNA decreased during SPC preparation, verifying the DNA damage along this preparation chain.

## 1. Introduction

In recent years, genetically modified (GM) soybean has become one of the most cultivated GM crops around the world. According to the International Service for the Acquisition of Agri-biotech Applications (ISAAA), the cultivation area of GM soybean was 95.9 million hectares in 2018, occupying 50% of the cultivation area of all GM crops [1]. Despite the environmental and economic benefits created by GM crops [2], the applications of GM crops in feed and food remain limited and controversial [3]. One of the major consumer concerns is the hazards of the potential horizontal transfer of transgenic DNA from GM crops to the human body [4]. As reported by Kharazmi, Bauer, Hammes, and Hertel [5], there are some potential risks from GM food, such as the potential horizontal transfer of recombinant DNA to gut-bacteria and food-associated bacteria. Since antibiotic- resistant genes have been used for GMO constructions, people are worried about the consequence of the spread of these genes to bacteria in human association. The only known way of enabling bacteria to acquire DNA from plants is transformation, and in actual fact it has been discovered that GM potato DNA can be naturally transferred to soil bacteria, such as *Pseudomonas stutzeri* and *Acinetobacter* sp. [6].

Nonetheless, some scholars reported that intact DNA molecules of certain sizes are necessary for the transformation of recombinant DNA to bacteria in foods [5]. Bauer, Weller, Hammes, and Hertel [7] discovered that for a transfer of functional genes from transgenic plants to bacteria, the minimal DNA fragment length should be at least 250 bp. Hence, degrading the transgenic DNA in GM crops during feed or food processing is a viable strategy to reduce the risks of GM crop-based products and improve the safety of GM products [8].

Previously, for sake of improving GMO detection methods and GM labelling legislation, a large number of works focusing on DNA degradation [9,10] and variations in transgenic content [11,12] along GM crop-based feed and food processing chain have been carried out. Whereas, few studies pay attention to the variations in the copy numbers and structural characteristics of transgenic DNA suffered from feed and food processing procedures, which are essential for the quantification of the DNA degradation ratio, the identification of the key step causing DNA degradation, and the clarification of DNA degradation mechanism during the processing of GM crop-based products remain undetermined [13].

As the main form of soybean protein, soybean protein concentrate (SPC) is vital to the feed and food industry. Considering the possibility of GM soybean being used as SPC, entering the human food chain, and thereby arousing consumer concern, we have recently demonstrated the distribution and degradation of DNA from GM soybean event GTS 40-3-2 during SPC preparation [14]. In order to further obtain the degradation ratio of the DNA and clarify the effects of SPC preparation on the structural characteristics of transgenic DNA, the copy numbers were absolutely quantified and the nanotopography and conformation were monitored in this research. The results do not only help identify key procedures inducing DNA degradation during SPC preparation, but also contribute to regulating the DNA degradation from the perspective of DNA structure variation, reducing the potential risk from GM soybeans, and providing a theoretical basis for the rational utilization of GM products in the future.

## 2. Materials and Methods

### 2.1. Materials

Seeds of pure GM soybean event GTS 40-3-2 containing the *cp*4 *epsps* coding sequence were generously provided by the Institute of Crop Sciences, Chinese Academy of Agricultural Sciences (Beijing, China).

### 2.2. SPC Preparation

**Figure 1:**
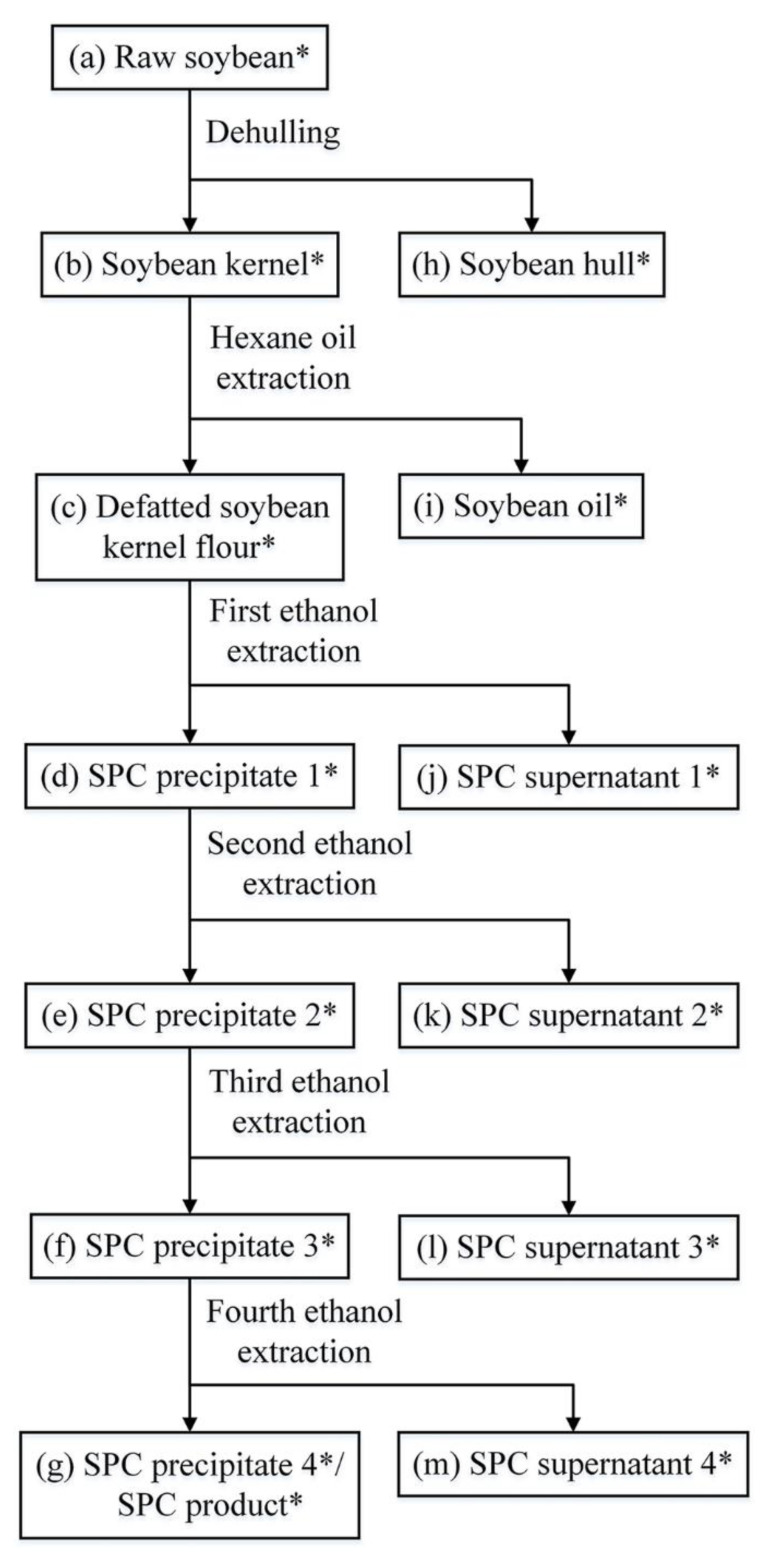
Flowchart of SPC preparation. Samples taken during the preparation line were marked with asterisks.

SPC was prepared according to the procedures described previously [14]. A total of 100 g of raw soybean was used, and samples from each preparation procedure, i.e., dehulling, defatting, the first to fourth ethanol extraction (Figure 1), were collected, lyophilized, weighed, and stored at 4 °C for further research. The ethanol cost for each soy washing batch was estimated to be 0.064 $/g sample.

### 2.3. Genomic DNA Isolation

According to Du et al. [14], DNAs from each sample were isolated, dispersed in DNase/RNase-Free Water (R1600, Solarbio Life Sciences, Beijing, China), and stored at −20 °C for further analysis. The UV spectrophotometry method was applied to assess the purity and quality of DNA extracts.

### 2.4. The Absolute Quantification of DNA by Droplet Digital PCR (ddPCR)

Droplet digital PCR (ddPCR) was performed using a QX200 Droplet Digital PCR System (Bio-Rad, Laboratories Inc., Hercules, CA, USA). Primers and probes for amplifying the 84 bp of the *lectin* target and the 87 bp of the *cp*4 *epsps* target are shown in Table 1 and were synthesized by Sangon Biotechnology (Shanghai, China).

The total volume of ddPCR reaction mixture was 20 μL, comprised of 10 μL of 2 × ddPCR Supermix for Probes (No dUTP, Bio-Rad), 0.9 μM of each primer, 0.25 μM of probe, and 1 μL of diluted template DNA. After loading 20 μL of the ddPCR mixture and 70 μL of Droplet Generation oil (Bio-Rad) into the sample well and oil wells of the DG8 cartridge (Bio-Rad), respectively, droplets were generated by using a QX200 Droplet Generator (Bio-Rad) [15]. Then, the generated droplets were transferred to a 96-well plate, sealed, and inserted into a T100 Thermal cycler (Bio-Rad) [16]. The amplification conditions were as follows: preincubation at 95 °C for 10 min; 40 cycles of denaturation at 94 °C for 30 s, annealing and extension at 60 °C for 60 s; followed by 10 min at 98 °C; and cooled to 4 °C finally. Next, the PCR plate was inserted into a QX200 Droplet Reader (Bio-Rad) for positive and negative droplets reading. Similarly to the data reported previously, the QuantaSoft software (Bio-Rad) was used for data acquisition and analysis [15], and the copy numbers of the *lectin* and *cp*4 *epsps* targets per reaction system were presented directly by this software [17].

Then, the total copy numbers of the *lectin* or *cp*4 *epsps* target from each sample could be calculated through Equation (1):

(1) $$\mathrm{Copy}\;\mathrm{number}\left(lectin/cp4\;epsps\right)=\frac{c(lectin/cp4\;epsps)\times D\times V\times m_{s}}{m_{0}}$$

where *c* (*lectin*/*cp*4 *epsps*) was the copy numbers of the *lectin* or *cp*4 *epsps* target per reaction system, *D* was the dilution factor [16] of the template DNA used for ddPCR (Table 2), *V* was the total volume of DNA extracts from the specific mass of each sample, *m_s_* was the total mass of each sample during SPC preparation, and *m*_0_ was the mass of the starting material for DNA isolation.

Then, the degradation ratio of the *lectin* or *cp*4 *epsps* target from each main product during SPC preparation could be calculated through Equation (2):

(2) $$\mathrm{Degradation}\;\mathrm{ratio}\left(lectin/cp4\;epsps\right)=[1-\frac{{\mathrm{Copy}\;\mathrm{number}}_{x}(lectin/cp4\;epsps)}{{\mathrm{Copy}\;\mathrm{number}}_{a}(lectin/cp4\;epsps)}]\times 100\%$$

where *x* ranged from b to g, representing soybean kernel, defatted soybean kernel flour, SPC precipitate 1, SPC precipitate 2, SPC precipitate 3, or SPC precipitate 4, respectively. Copy number*_x_* (*lectin*/*cp*4 *epsps*) was the total copy numbers of the *lectin* or *cp*4 *epsps* target from soybean kernel, defatted soybean kernel flour, or SPC precipitate 1–4. Copy number*_a_* (*lectin*/*cp*4 *epsps*) was the total copy numbers of the *lectin* or *cp*4 *epsps* target from raw soybean.

### 2.5. Atomic Force Microscopy (AFM) Tests

Samples used for AFM tests were prepared according to Chammas, Bonass, and Thomson [18] with slight modifications: 5 ng/μL of DNA was diluted in an equal volume of imaging buffer containing 4 mM of Tris-HCl (pH 7.5, Solarbio Life Sciences, Beijing, China) and 4 mM of MgCl_2_. Then, 20 μL of the homogeneous mixture was dropped onto freshly cleaved mica (Ted Pella, Inc., Redding, CA, USA) and incubated at room temperature to ensure adsorption of the DNA. After 10 min of incubation, the mica was rinsed with 1 mL of double-distilled water three times, and dried in air overnight [19].

AFM imaging was completed using an MFP-3D Infinity AFM (OXFORD Instruments Asylum Research, Santa Barbara, CA, USA). The tapping mode was used in air at 25 °C, the scan rate was 1.00 Hz, and the scan size was 4 × 4 μm.

### 2.6. Circular Dichroism (CD) Tests

The CD spectra of DNA from the main products collected along the SPC preparation chain were recorded from 195 to 325 nm using a JASCO J-810 circular dichroism instrument (JASCO International Co. Ltd., Tokyo, Japan). The temperature was 25 °C, the bandwidth was 1.0 nm, and the concentration of each sample was 300 ng/μL [20].

### 2.7. Fluorescence Spectrometry Tests

The fluorescence intensity analysis was performed using a FLS 1000 Transient Steady-state Fluorescence Spectrometer (Edinburgh Instruments Ltd., Edinburgh, UK). The total volume of each sample for detection was 2.5 mL, containing 2 ng/μL of DNA, 2.5 mM of Tris-HCl (pH 7.5, Solarbio), and 4 ng/μL of EB (Sangon Biotechnology, Shanghai, China). The fluorescence spectra were acquired by emission excitation at 500 nm, and scanning from 550 to 800 nm [21].

### 2.8. Statistical Analysis

Each test was performed in triplicate. SPSS 21.0 was used for statistical analysis. One-way analysis of variance (ANOVA) was conducted to compare the experimental results, and a value of *p* < 0.05 was considered significant. DdPCR results were presented as the means ± standard deviations (SDs). CD spectra and fluorescence spectra results were reported as mean values according to Li, Wang, Zhao, Ouyang, and Wu [22]. The figures were drawn using Origin 8.5.

## 3. Results and Discussion

### 3.1. Variations in Copy Numbers of the Lectin and cp4 Epsps Targets during SPC Preparation

Dehulling, defatting, and ethanol extraction are the main procedures during SPC preparation, and involve mechanical treatment, thermal treatment, and chemical treatment, etc. After collecting and weighing all of the main products and by-products, the mass of each product was found to conform to the law of mass conservation (Table 2). For example, after dehulling, the total mass of soybean kernel and soybean hull (98.88 g) was lower than or equal to the mass of raw soybean (100 g). These results also provided data support for the calculation of the total copy numbers and degradation ratios of the DNA from each product.

Droplet digital PCR (ddPCR) is an emerging quantitative PCR technique, accomplishing the absolute quantification of target DNA without reference material [23]. Because the detection and quantification limits are low, and the repeatability, fidelity, and robustness perform well, ddPCR is deemed to be reliable and applicable in GM organisms’ quantification [16,24]. Table 2 demonstrates the ddPCR amplification results of the *lectin* and *cp*4 *epsps* targets. Before the amplification, DNAs from raw soybean, soybean hull, soybean kernel, defatted soybean kernel flour, and SPC precipitate 1–4 were diluted 5–40 times. The copy numbers of the *lectin* or *cp*4 *epsps* target per reaction system (*c*), as well as the dilution factor (*D*), the total volume of DNA extracts (*V*), the total mass of each sample during SPC preparation (*m_s_*), and the mass of starting material for DNA isolation (*m_0_*) are presented in Table 2. As the relative standard deviations (RSDs) for all of the samples were less than 25%, which is the acceptance limit, the ddPCR results were deemed reliable [23]. According to Equation (1), the total copy numbers of the *lectin* and *cp*4 *epsps* targets from each sample were obtained.

Table 2 shows that the copy numbers of both targets from soybean kernel, defatted soybean kernel flour, and SPC precipitate 1–4 were 1.04 × 10^7^–1.18 × 10^9^, while those from soybean hull, soybean oil, and SPC supernatant 1–4 were much lower (1.27 × 10^3^–2.39 × 10^7^). Therefore, the majority of the *lectin* and *cp*4 *epsps* targets were distributed in the main products after dehulling, defatting, and the first to fourth ethanol extraction, which was expected from the distribution of DNA discussed previously [14]. As proteins were also distributed more in the main products than in the by-products along the SPC preparation chain, it was deduced that the distribution of DNA was related to the distribution of protein.

Furthermore, the degradation of the *lectin* and *cp*4 *epsps* targets was also discovered through the comparison of total copy numbers before and after each procedure. For example, the total copy numbers of the *lectin* and *cp*4 *epsps* targets from 100 g of raw soybean were 1.06 × 10^9^ and 1.29 × 10^9^, respectively. Yet, after dehulling, the sum of the total copy numbers of each target from 86.36 g of soybean kernel and 12.52 g of soybean hull decreased to 9.46 × 10^8^ and 1.20 × 10^9^, respectively. Based on Equation (2), the degradation ratios of the *lectin* and *cp*4 *epsps* targets from the main products after each procedure were calculated. During SPC preparation, the degradation ratios of both targets clearly increased from around 10% in soybean kernel to 99% in SPC product. Meanwhile, the degradation ratios of both targets from defatted soybean kernel flour were 40.00–49.30% higher than those from soybean kernel, and the degradation ratios of both targets from SPC precipitate 1 were 36.88–41.35% higher than those from defatted soybean kernel flour. Hence, defatting and the first ethanol extraction were considered to be key procedures causing DNA degradation. The degradation ratios of both targets reached above 94% when the first ethanol extraction was completed.

As we know, GM technology is one of the available means to cope with the future foodstuff crisis. Many countries have planted GM crops and approved GM foods to circulate in the market [25]. However, the utilization of GM crops is still limited and controversial mainly because consumers fear the risks of the transformation of recombinant DNA from GMOs to bacteria in the gastrointestinal tract and to food destined for human consumption [4]. Some reports stated that degradation of the exogenous genes would contribute to depriving them of their original gene functions, and thereby alleviate the potential hazards of GM crops and improve the safety of GM crop-based products [7]. In this research, the degradation ratio of the *lectin* and *cp*4 *epsps* targets increased sharply after defatting and the first ethanol extraction, implying the important role of these two methods in DNA degradation. Therefore, the parameters used in defatting and ethanol extraction could be adjusted to regulate DNA degradation during SPC preparation.

Existing studies showed that the transgenic DNA from GMOs could be damaged through grinding, high temperature, ultrasound, microwave, extreme pH, etc. [26]. However, the effect of ethanol extraction on transgenic DNA degradation has not been taken seriously. Ethanol is an environmentally friendly and recyclable organic solvent with low toxicity, heat capacity, latent heats of crystallization and evaporation, boiling point, and density and lower energy consumption. Ethanol extraction is an inexpensive, simple, and efficient method that has been applied for refining substances (such as proteins, anthocyanins, isoflavones, and phospholipids) from oilseeds, cereal grains, algae, and wood pulp, etc [27]. Hence, ethanol extraction promises to be an alternative way to promote DNA degradation during feed and food production in the future.

### 3.2. Variations in the Nanotopography of DNA during SPC Preparation

**Figure 2:**
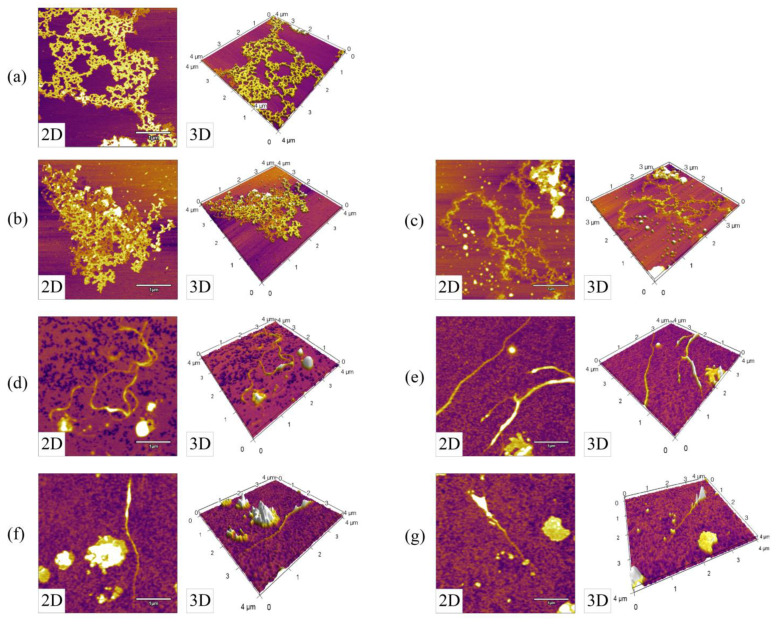
2D and 3D views of AFM images of DNA from (**a**) raw soybean, (**b**) soybean kernel, (**c**) defatted soybean kernel flour, (**d**) SPC precipitate 1, (**e**) SPC precipitate 2, (**f**) SPC precipitate 3, (**g**) SPC precipitate 4/SPC product.

Atomic force microscopy (AFM) is an attractive tool for DNA microstructure characterization due to the high resolution and simple sample preparation procedures [28]. Figure 2 illustrates the 2D and 3D views of the nanotopography of DNA from the main products collected during SPC preparation. The same amount of DNA was loaded onto the mica for better comparison. It was observed that long and consecutive DNA chains were thick and dense simultaneously in raw soybean and soybean kernel (Figure 2a,b). In contrast, after defatting, denature of the DNA occurred to some extent, as evidenced by the thinner and sparser DNA chain in defatted soybean kernel flour (Figure 2c). After ethanol extraction, the DNA chains continued to become sparser and shorter in the SPC precipitate 1–4 (Figure 2d–g), confirming the degradation of DNA described in Section 3.1. Meanwhile, some DNA aggregations were generated, and were similar to those observed by Wang, Ran, Man, and Yang [29] in a high concentration of ethanol and by Castellanos [30] under X-ray irradiation.

### 3.3. Variations in the Conformation of DNA during SPC Preparation

Circular dichroism (CD), a phenomenon produced by the interaction of chiral molecules with circularly polarized electromagnetic rays, is widely used for mapping the conformations of DNA, including the B-type, A-type, and Z-type, etc. [31]. Both of the B-DNA and A-DNA are right-handed helixes exhibiting positive and negative bands at different wavelengths. For B-DNA, the positive band appears at around 260–280 nm corresponding to base stacking, and the negative band appears at around 245 nm corresponding to helicity [32]. However, for A-DNA, positive and negative bands appear at around 260 nm and 210 nm, respectively [31]. The differences between the conformations of DNA that are reflected by CD spectra are generally related to base compositions, (G+C) contents [33], or temperatures [34], etc.

**Figure 3:**
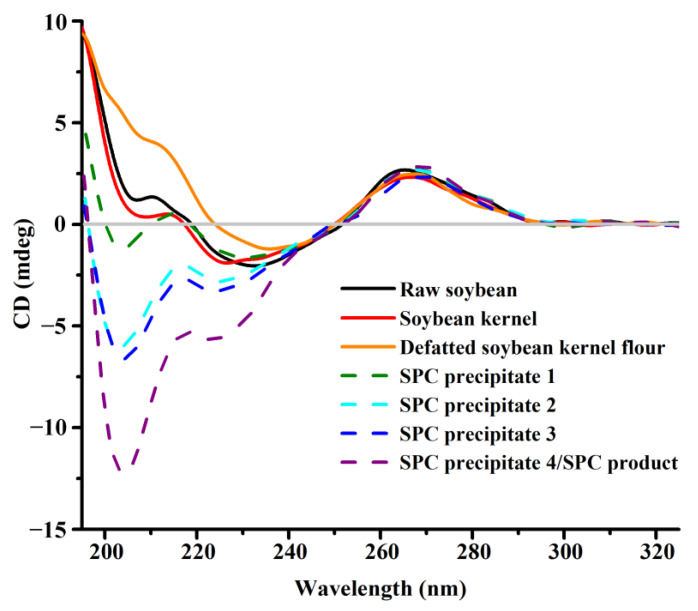
CD spectra of DNA from main products during SPC preparation.

Figure 3 presents the CD spectra of DNA from the main products during SPC preparation. For raw soybean, soybean kernel, and defatted soybean kernel flour, a positive band at around 265 nm and a negative band at around 240 nm were observed. This implied that the DNAs from these three samples were right-handed B-type double helixes, consistent with the basic conformation of DNA from eukaryotes [31]. As there was no apparent difference between the CD spectra of DNA from raw soybean and that from soybean kernel, dehulling was considered to have little effect on the conformation of soybean DNA. On the contrary, after defatting, the negative band with a maximum at about 205 nm disappeared, indicating a weaker helicity of the DNA from defatted soybean kernel flour compared with that from soybean kernel [32], which explained the denature and degradation of the DNA described in Section 3.1 and Section 3.2.

Then, after the first to fourth ethanol extraction, a new negative band at around 205 nm was observed in the CD spectra of DNAs from SPC precipitate 1–4 (Figure 3). This result demonstrated the conformation transition of DNA from B-type to A-type during ethanol extraction [31]. Similar B-to-A type transition of DNA induced by a water-ethanol mixture has been reported previously [35]. The phenomenon was attributed to the low dielectric constant of water-nonelectrolyte solutions, that affected the electrostatic interaction and actuated the transition of the DNA conformation consequently [36].

As is known, B-DNA and A-DNA adopt the right-handed helical structure formed by two antiparallel polynucleotide chains around a central axis. There are spiral-shaped grooves between the polynucleotide chains, one of which is shallow and narrow and is called the minor groove, while the other is large and wide and called the major groove [37]. Viewed from the top to the bottom of the DNA along the helix axes, the base plane of B-DNA is perpendicular to the longitudinal axis with the axes passing through the hydrogen bond between the two polynucleotide chains. Compared with B-DNA, the base pairs of A-DNA are inclined towards the edge of the DNA double helix, with an inclination of 20°. Hence, the minor groove of the A-DNA is shallower and wider than that of B-DNA, while the major groove is deeper and narrower. Cheatham, Crowley, Fox, and Kollman [38] pointed out that ethanol would destroy the spine of hydration in the minor groove of DNA and affect the presence of ion-mediated interhelical bonds and extensive hydration across the major groove of DNA. Hence, the free energy of A-DNA was lower than that of B-DNA in high concentrations of ethanol, contributing to higher stability. As described in Section 3.1 and Section 3.2, DNA degradation occurred during ethanol extraction. We speculated that the DNA degradation was related to the transition of DNA conformation. When high concentrations of ethanol were added to the defatted soybean kernel flour, the free energy of B-DNA was high with low stability and tended to transform into A-DNA with lower free energy. Due to the formations of thermal instability sites and the breakages of hydrogen bonds, depurination, deamination, or other hydrolysis reactions of DNA would occur during the conformation transition causing the DNA degradation and the decrease in the copy numbers of targeted DNAs as a result [39].

Furthermore, the conformation transition of the DNA could reflect the variations in base composition, which is conducive to predicting the DNA degradation sites [40]. According to Kypr et al. [31], DNA fragments such as poly[d(A)]·poly[d(T)], never adopt the A-type conformation, while those rich in (G+C) tend to exhibit A-type conformation even in an aqueous solution [41]. Therefore, it was inferred that most of the DNA degradation during ethanol extraction occurred at (A+T) sites owing to the lower thermal stability compared with (G+C) sites [12,33].

### 3.4. Variations in the Fluorescence Emission Spectra of DNA during SPC Preparation

Fluorescence spectrometry is another way to characterize the secondary structure of DNA composed of anionic polyelectrolyte and phosphate groups [42]. Ethidium bromide (EB), a cation dye that is able to intercalate to the base pairs of DNA, is always employed as a molecule probe in DNA fluorescence spectrometry tests [43].

**Figure 4:**
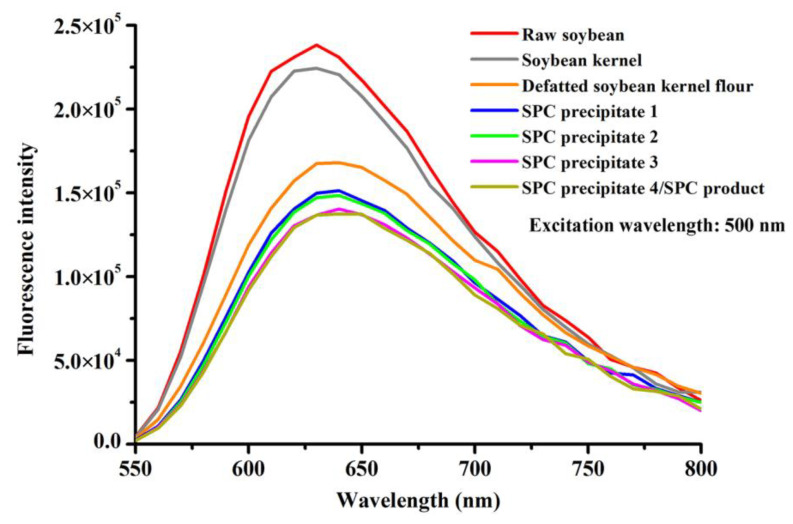
Fluorescence spectra of DNA from the main products during SPC preparation.

Figure 4 presents the fluorescence emission spectra of soybean DNA bind with EB during SPC preparation. The maximums of fluorescence bands at around 625 nm were observed for all of the seven samples, in agreement with the fluorescence emission spectra of calf thymus DNA binding with EB [21]. For raw soybean and soybean kernel, there was a difference between the fluorescence emission spectra of DNA with relative high intensity values compared to the intensity values of DNAs from the other samples. Hence, when the CD and nanotopography results were also taken into account, the double-strand helix DNA molecules were considered to be intact before defatting. However, as the SPC preparation procedures continued, the intensity of fluorescence at its maximum in DNA molecules tended to decrease, confirming the variations in the conformation of DNA and the degradations of DNA that may contribute to the reduction of the double helix [43]. Damage to double-strand helix DNA has been found under gamma radiation previously, as evidenced by the fact that the fluorescence intensity of calf thymus DNA tended to decrease when the radiation dose increased from 0 Gy to 3756 Gy [21]. As reported by Cai and Cherian [44], this DNA damage may be induced by strand breaks, base liberation, and base oxidation, etc.

## 4. Conclusions

In this study, ddPCR was applied for absolute quantification of DNA from GM soybean event GTS 40-3-2 during SPC preparation. AFM, CD, and fluorescence spectrometry were applied to reflect the nanotopographies and secondary structures of DNA from the main products. Along the preparation chains, copy numbers of the *lectin* and *cp*4 *epsps* targets decreased by about two orders of magnitude. Among all of the procedures included in SPC preparation, dehulling had little influence on DNA degradation, whereas after defatting, the degradation ratios of the *lectin* and *cp*4 *epsps* targets reached 53.02% and 57.83%, respectively. The DNA chains became thinner, and the helicity of the DNA decreased. Then, shorter DNA chains and the B-to-A transition of DNA conformation were observed after the first ethanol extraction. The degradation ratios of both targets increased by 41.35% and 36.88%, respectively. Besides, further degradation of DNA occurred during the following ethanol extractions. The total copy numbers of both targets decreased to 1.04 × 10^7^ and 1.18 × 10^7^, respectively, in the final SPC product. These results not only contribute to providing a theoretical basis and available ways to promote the degradation of transgenic DNA and to reducing the potential risk from GM crops, but also help to utilize GM feed and food products healthily and rationally. As it has been identified that defatting and the first ethanol extraction were key procedures in inducing DNA degradation during SPC preparation, the parameters used in these procedures will be adjusted to facilitate DNA degradation in our upcoming research.

## Figures and Tables

**Table 1:** Primers and probes used in ddPCR.

DNA Target	Primer Sequence (5′-3′)		Amplicon Size (bp)	Location of Amplified Fragment in GenBank Sequence
*lectin* (K00821)	F:	GCTTCGCCGCTTCCTTC	84	1269–1352
	R:	TTGGTGCGAGAAAGAAGGC		
	Probe:	FAM-TTCACCTTCTATGCCC-BHQ-1		
*cp*4 *epsps* (AB209952)	F:	CCTCCGATGATCGACGAAT	87	1476–1562
	R:	GAGTTCTTCCAGACCGTTCATC		
	Probe:	FAM-CCGATTCTCGCTGTC-BHQ-1		

**Table 2:** ddPCR results of the *lectin* and *cp*4 *epsps* target from each sample collected during SPC preparation.

Samples			*m_s_* (g)	*m*_0_ (g)	*V* (μL)	*lectin* Target					*cp*4 *epsps* Target				
						*D*	*c* (Copies/μL)	Copy Number * (Copies)	RSD%	Degradation Ratio (%)	*D*	*c* (Copies/μL)	Copy Number * (Copies)	RSD%	Degradation Ratio (%)
Main products	a	Raw soybean	100.00	0.1	50	40	528 ± 39	(1.06 ± 0.08) × 10^9 A^	7%	/	10	2570 ± 167	(1.29 ± 0.08) × 10^9 A^	6%	
	b	Soybean kernel	86.36	0.1	50	40	534 ± 20	(9.22 ± 0.35) × 10^8 B^	4%	13.02 ± 3.31	10	2740 ± 53	(1.18 ± 0.02) × 10^9 B^	2%	8.53 ± 1.78
	c	Defatted soybean kernel flour	60.20	0.1	50	40	414 ± 15	(4.98 ± 0.18) × 10^8 C^	4%	53.02 ± 1.72	10	1806 ± 60	(5.44 ± 0.18) × 10^8 C^	3%	57.83 ± 1.40
	d	SPC precipitate 1	45.23	0.1	50	10	264 ± 12	(5.97 ± 0.27) × 10^7 D^	5%	94.37 ± 0.26	10	302 ± 15	(6.83 ± 0.34) × 10^7 D^	5%	94.71 ± 0.27
	e	SPC precipitate 2	41.46	0.1	50	10	196 ± 12	(4.06 ± 0.25) × 10^7 DE^	6%	96.17 ± 0.24	10	280 ± 16	(5.80 ± 0.32) × 10^7 D^	6%	95.50 ± 0.25
	f	SPC precipitate 3	38.89	0.1	50	5	158 ± 4	(1.54 ± 0.04) × 10^7 E^	3%	98.55 ± 0.04	5	274 ± 18	(2.66 ± 0.18) × 10^7 DE^	7%	97.94 ± 0.14
	g	SPC precipitate 4/SPC product	35.28	0.1	50	5	118 ± 9	(1.04 ± 0.08) × 10^7 E^	7%	99.02 ± 0.07	5	134 ± 19	(1.18 ± 0.17) × 10^7 E^	14%	99.09 ± 0.13
By-products	h	Soybean hull	12.52	0.1	50	10	380 ± 18	(2.38 ± 0.11) × 10^7 DE^	5%	/	10	382 ± 28	(2.39 ± 0.18) × 10^7 DE^	7%	/
	i	Soybean oil	25.58	9.3	30	1	46 ± 11	(3.80 ± 0.92) × 10^3 E^	24%	/	1	15.4 ± 3.1	(1.27 ± 0.25) × 10^3 E^	20%	/
	j	SPC supernatant 1	7.80	0.1	20	1	32 ± 7	(4.99 ± 1.12) × 10^4 E^	22%	/	1	7.2 ± 1.8	(1.12 ± 0.28) × 10^4 E^	25%	/
	k	SPC supernatant 2	3.17	0.1	20	1	58 ± 9	(3.68 ± 0.58) × 10^4 E^	16%	/	1	6.6 ± 1.4	(4.18 ± 0.89) × 10^3 E^	21%	/
	l	SPC supernatant 3	1.66	0.1	20	1	42 ± 8	(1.39 ± 0.27) × 10^4 E^	19%	/	1	15.0 ± 2.6	(4.98 ± 0.86) × 10^3 E^	17%	/
	m	SPC supernatant 4	0.86	0.1	20	1	42 ± 5	(7.22 ± 0.91) × 10^3 E^	13%	/	1	9 ± 1	(1.55 ± 0.17) × 10^3 E^	11%	/

* The different superscript capital letters in each column indicate significant difference (*p* < 0.05).

## Data Availability

All related data and methods are presented in this paper. Additional information is available on request from the correspondence author. Inquiries should be addressed to the corresponding author.
